# ZipHiC: a novel Bayesian framework to identify enriched interactions and experimental biases in Hi-C data

**DOI:** 10.1093/bioinformatics/btac387

**Published:** 2022-06-09

**Authors:** Itunu G Osuntoki, Andrew Harrison, Hongsheng Dai, Yanchun Bao, Nicolae Radu Zabet

**Affiliations:** Department of Mathematical Sciences, University of Essex, Colchester CO4 3SQ, UK; Statistics, Modelling and Economics Department, UK Health Security Agency, London NW9 5EQ, UK; Department of Mathematical Sciences, University of Essex, Colchester CO4 3SQ, UK; Department of Mathematical Sciences, University of Essex, Colchester CO4 3SQ, UK; Department of Mathematical Sciences, University of Essex, Colchester CO4 3SQ, UK; School of Life Sciences, University of Essex, Colchester CO4 3SQ, UK; Blizard Institute, Barts and The London School of Medicine and Dentistry, Queen Mary University of London, London E1 2AT, UK

## Abstract

**Motivation:**

Several computational and statistical methods have been developed to analyze data generated through the 3C-based methods, especially the Hi-C. Most of the existing methods do not account for dependency in Hi-C data.

**Results:**

Here, we present ZipHiC, a novel statistical method to explore Hi-C data focusing on the detection of enriched contacts. ZipHiC implements a Bayesian method based on a hidden Markov random field (HMRF) model and the Approximate Bayesian Computation (ABC) to detect interactions in two-dimensional space based on a Hi-C contact frequency matrix. ZipHiC uses data on the sources of biases related to the contact frequency matrix, allows borrowing information from neighbours using the Potts model and improves computation speed using the ABC model. In addition to outperforming existing tools on both simulated and real data, our model also provides insights into different sources of biases that affects Hi-C data. We show that some datasets display higher biases from DNA accessibility or Transposable Elements content. Furthermore, our analysis in *Drosophila melanogaster* showed that approximately half of the detected significant interactions connect promoters with other parts of the genome indicating a functional biological role. Finally, we found that the micro-C datasets display higher biases from DNA accessibility compared to a similar Hi-C experiment, but this can be corrected by ZipHiC.

**Availability and implementation:**

The R scripts are available at https://github.com/igosungithub/HMRFHiC.git.

**Supplementary information:**

Supplementary data are available at *Bioinformatics* online.

## 1 Introduction

Distant regulatory elements and their target genes are often separated by large genomic distances. In order for the regulatory element to activate a target gene, they need to come in 3D proximity (Bonev and Cavalli, 2016; Hua *et al.*, 2021). This indicates that the spatial organization of the genome is intimately related to genome regulation and a better understanding of the 3D organization of the genome is important in disentangling the contribution of different factors to gene regulation. One of the recently developed genome-wide proximity ligation assay is the Hi-C technique (Lieberman-Aiden *et al.*, 2009), which is a chromosome conformation capture (3C)-based method. Hi-C is able to detect interactions (short-range and long-range) within and between chromosomes at high resolutions. While in mammalian systems, resolutions of 5 Kb have been achieved (Rao *et al.*, 2014), in smaller genomes, such as *Drosophila*, sub-kilobase pair resolutions were obtained from Hi-C experiments (Chathoth and Zabet, 2019; Cubenãs-Potts *et al.*, 2017; Eagen *et al.*, 2017). In addition, datasets generated by Hi-C are highly reproducible between replicates and often highly conserved between tissues (Ghavi-Helm *et al.*, 2014). Recent technological advances have pushed the resolution of conformation capture methods to base pair resolution in mammalian systems (Hua *et al.*, 2021).

The data generated by a Hi-C experiment can be represented as a matrix of contact frequencies between pairs of regions along the genome. These matrices are associated with biases (Yaffe and Tanay, 2011), such as the restriction fragment length, GC content of trimmed ligation junctions and mappability, but many additional factors may also contribute to the contact counts. Correcting for these biases is important and there have been several methods being proposed that take these biases into account (Hu *et al.*, 2013; Imakaev *et al.*, 2012; Servant *et al.*, 2015; Yaffe and Tanay, 2011).

The Iterative Correction and Eigenvector decomposition (ICE) has been the most widely used method to account for biases associated with the Hi-C data, due to its simplicity and being parameter-free by assuming equal visibility across all regions of the genome (Imakaev *et al.*, 2012). This equal visibility assumption considers that all regions can be probed by the method with same probability. However this assumption is not always true, because the visibility of areas could vary (Imakaev *et al.*, 2012; Servant *et al.*, 2015). In addition, ICE is computationally intensive because the Hi-C interaction matrix is of size $O(N^{2})$, where *N* is the number of genomic regions.

The study of Rao *et al.* (2014) generated one of the highest-resolution maps of the 3D organization of the human genome using an *in situ* Hi-C to probe the 3D architecture of genomes for DNA–DNA proximity ligation in intact nuclei. This has revealed that the human genome is organized into sub-compartments globally and contains about 10 000 chromatin loops (Rao *et al.*, 2014). To account for biases in Hi-C data, Rao *et al.* (2014) adopts the matrix-balancing proposed in Knight and Ruiz (2013). In particular, peaks are called only when a pair of regions of the genome shows elevated contact frequency relative to the local background; i.e. peaks are called when the peak pixel is enriched as compared to other pixels in its neighbourhood.

Other methods take into account potential dependence among pairs of regions of the genome (Jin *et al.*, 2013). To accurately identify the chromatin interactions and loops with high sensitivity and resolution, they used data filtering techniques based on the strand orientation of Hi-C paired-end reads. This also allows detection of short genomic distance interactions between restriction fragments and their analysis shows the effects of GC content and mappability on the observed contact frequency. Interestingly, there seems to be a linear relationship between average trans-contact frequency and mappability (Jin *et al.*, 2013).

Loci that are in close 1D proximity to each other often interact with the same distal regions. This suggests that these loci are part of a region that make a 3D contact with the distal region. Some of the existing methods are based on one-dimensional calling approaches, which do not consider useful information that can be gained using the two-dimensional approach. The first method to take into account the spatial dependency of Hi-C is the HMRFBayesHiC algorithm (Xu *et al.*, 2016b). In particular, HMRFBayesHiC models the neighbouring regions in the context of a two-dimensional contact matrix generated from Hi-C. This algorithm assumes that not all peaks will have similar strength and clustering patterns. Nevertheless, it also involves having prior information about the expected count frequency distribution to account for biases, which is often unknown. One of the biggest shortcomings of this approach is that it is computationally intensive and chromosome wide computations, even in smaller genomes, are not feasible.

FastHiC is a novel hidden Markov random field (HMRF)-based peak caller to detect long-range chromosomal interactions from Hi-C data (Xu *et al.*, 2016a). The FastHiC method is based on the HMRFBayesHiC (Xu *et al.*, 2016b) and uses simulated field approximation, which approximates the joint distribution of the hidden peak status by a set of independent random variables. In particular, FastHiC approximates the Ising distribution by a set of independent random variables, enabling tractable computation of the normalizing constant in the Ising model. Despite this improvement in computation time, FastHiC is still computationally intensive and chromosome wide calculations are still computationally challenging.

FitHiC2 is an extended and improved version of the Fit-Hi-C (Ay *et al.*, 2014) which incorporates various new computational modules and pre-/post-processing utilities (Kaul *et al.*, 2020). The FitHiC2 is designed to compute statistical confidence estimates to Hi-C counts by fitting a cubic smoothing spline to the average genomic distance and contact probabilities in Hi-C datasets to learn a continuous function that relates the average genomic distance and contact probabilities (Kaul *et al.*, 2020). Despite the simplicity of FitHiC2, it fails to consider the possibility of spatial dependency in the Hi-C data.

Another recently developed method for the detection of chromatin interactions from Hi-C data is the HiC-ACT which uses the Cauchy test (Lagler *et al.*, 2021). HiC-ACT addresses the possible spatial dependency ignored in the FitHiC2 method, but it is more computationally intensive compared with the FitHiC2, (Lagler *et al.*, 2021). However, one of the limitations of the HiC-ACT method is that it is a post-processing method, which only requires bin identifiers and probabilities generated from other methods rather than the raw Hi-C data.

Finally, all these previous methods often classify the observations into only two classes: non-random contacts (peaks) and random contacts (noise). Nevertheless, it is possible to have more than two classes due to the nature of the Hi-C approach. For example, a non-random contact may have similar bias information to a random contact, which may lead to a misclassification of this pair of regions by the existing methods.

In this article, we present ZipHiC, a hidden Markov random field-based Bayesian approach to identify significant interactions in Hi-C data. This new model addresses several issues with current models. First, we improve on existing methods by introducing the dependency of neighbouring regions in the two-dimensional space and adopt the Approximate Bayesian Approach (ABC) to deal with the intractable normalizing constant in the Potts model, a Markov random field-based model (Wu, 1982). Second, our model is computationally tractable and can be applied chromosome wide. Third, the number of classes under consideration can be naturally extended to more than two. We focus our analysis on intra-chromosomal interactions due to the fact that about 95% of non-random interactions are found within chromosomes (Jin *et al.*, 2013; Xu *et al.*, 2016b). Most importantly, we use ZipHiC to model Hi-C contact maps in *Drosophila* cells and human cells and explore biases introduced by GC content, transposable elements (TEs) and DNA accessibility. Finally, we also model micro-C data in human ES cells and compare it to a similar Hi-C dataset in terms of the identified significant contacts and biases.

## 2 Materials and methods

### 2.1 Ziphic

#### 2.1.1 Notations

ZipHiC uses the contact matrix between pairs of bins generated from Hi-C experiments. Let *y_ij_*, 0≤i<j≤N denote the observed contact frequency between bin *i* and bin *j* in *N* total bins and *D_ij_* represent the genomic distance between bin *i* and bin *j*. Let *GC_ij_* represent the average percentage of Guanine and Cytosine, *TE_ij_* represent the average number of transposable elements (TEs) and *ACC_ij_* represent the average DNA accessibility score in bins *i* and *j*. For simplicity, we use s={i,j} to denote the interaction pair of bins *i* and *j* and use *D_s_*, *GC_s_*, *ACC_s_* and *TE_s_* to denote the observation value for interaction *s*.

#### 2.1.2 Mixture model for data

We use the *K*-component mixture density to model our data *y_ij_*, where the first component is a zero-inflated Poisson (ZIP) distribution for noise (see below), while the other components follow Poisson distributions:

(1) $$f(y_{ij})=\alpha_{1}\text{ZIP}(\tau ,\lambda_{ij}^{\left(1\right)})+\sum_{k=2}^{K}\alpha_{k}P\mathrm{ois}(\lambda_{ij}^{\left(k\right)})$$

where *τ* is the probability of extra zeros, $\lambda_{ij}^{\left(k\right)}$ is the mean of the *k*th component. *α_k_* is unknown percentage of *k*th component subject to the constraint $\sum_{k=1}^{K}\alpha_{k}=1$.

The above mixture model can be interpreted via a latent variable framework. We introduce the latent variable $z_{ij}=k,k=1,2,\ldots ,K$, where $z_{ij}=k$ means that *y_ij_* follows the distribution of component *k*. Furthermore, $\lambda_{ij}^{\left(k\right)}$ represents the mean interaction of bins *i* and *j* if it is from the *k*th component. The unknown number of mixture components *K* makes the framework more flexible for different scenarios. Our model accommodates increasing from 2 components to any number of components. Nevertheless, in this article, we found that *K* = 3 is sufficient to model the data and, thus, we did not use more than three components in our analysis.

Due to the fact that the Hi-C contact map displays excess zero-counts and that the mean and variance are not the same, we assume that the noise follows a ZIP distribution rather than a Poisson distribution. In particular, a ZIP distribution has the mean (1−τ)λ and variance λ(1−τ)(1+τλ). Furthermore, we assume that the sources of biases can be corrected by modelling $\lambda_{s}^{\left(k\right)}$ with s={i,j},k=1,2,…,K as

(2) $$\begin{array}{c} \log(\lambda_{s}^{\left(k\right)})=\beta_{0}^{\left(k\right)}+\beta_{1}^{\left(k\right)}\;\text{log}(D_{s})+\beta_{2}^{\left(k\right)}\;\text{log}(GC_{s})+\beta_{3}^{\left(k\right)}\;\text{log}(TE_{s})\\ +\beta_{4}^{\left(k\right)}\;\text{log}(Acc_{s}) \end{array}$$

#### 2.1.3 Potts model

To introduce the spatial dependency, our method utilizes the HMRF for the hidden components. The HMRF is a generalization of the hidden Markov model (HMM). The HMRF has been widely used in areas such as image analysis (Zhang *et al.*, 2001), gene expression data analysis (Wei *et al.*, 2008) and a population genetics study (François *et al.*, 2006). We adopt the Potts model (Wu, 1982) based on a Markov random field which provides a flexible way to model spatially dependent data as our prior for the latent variable *z_s_*. The latent variable ***z*** adopting the Potts model is written as

(3) $$p(z|\gamma )=\frac{1}{C(\gamma )}\text{exp}(\gamma \sum_{\left(s∼t\right)}\delta_{z_{s}z_{t}})$$

where $\delta_{z_{s}z_{t}}$ is the Kronecker symbol which takes the value 1 when *z_s_* = *z_t_* and 0 otherwise. Label *t* defines the neighbouring bin pairs of *s*, i.e. s∼t means *s* and *t* are neighbours in the Hi-C matrix. The set of latent variables *z_ij_* are modelled as a 2-dimensional HMRF, so the latent variable *z_s_* depends on the status of the neighbours of $s=\{i,j\},\;N_{s}=\{(i+1,j),(i-1,j),(i,j+1),(i,j-1)\}$. The neighbouring $\sum_{\left(s,t\right)}\delta_{z_{s}z_{t}}$ can be interpreted as the sum of the influence of neighbours of *s*. Here, *γ* is a non-negative interaction parameter, with value 0 resulting in an independent uniform distribution on *z_ij_*. Larger values of *γ*, such as *γ* = 1, corresponds to a high level of spatial interaction, and the probability of pairs of neighbours being in the same component is very high. C(γ) is the normalizing constant, also known as the partition function, which is written as

(4) $$C(\gamma )=\sum_{z}\;\text{exp}\;(\gamma \sum_{\left(s∼t\right)}\delta_{z_{s}z_{t}})$$

where ∑ _*z*_ indicates the summation over *z_s_* at all interactions *s* and it depends on the interaction parameter *γ*. The normalizing constant is computationally intractable in higher order. To overcome this complication, methods such as the likelihood-free approach can be used. Here, we use the Approximate Bayesian Computation model (ABC) (Beaumont *et al.*, 2002).

#### 2.1.4 Approximate Bayesian Computation model (ABC)

With a given dataset $Y=(y_{1},y_{2},\ldots ,y_{n})$ that is associated with the models in Equations (1), (2) and (3), the ABC algorithm (Beaumont *et al.*, 2002) used here can be described as follows.

1. Simulate an initial value *γ*_0_ from the prior distribution $\pi_{0}(\gamma )$;
2. Generate a parameter value from the posterior distribution $\pi (\gamma |Y)\propto \pi_{0}(\gamma )p(z|\gamma )$;
3. A new value of $\gamma^{*}$ and $y^{*}$ is simulated jointly from (1), (2) and (3);
4. Compute the absolute genomic distance or Euclidean distance *d* between the simulated data and the observed data;
5. Fix a tolerance *ϵ* or use an empirical quantile of $d(y^{*},y)$ which often corresponds to 1% quantile (Beaumont *et al.*, 2002)
6. Accept $\gamma^{*}$ if the absolute genomic distance is less than *ϵ*, otherwise reject and start from step 1 again.

#### 2.1.5 Bayesian inference

To infer parameters, we adopt the Bayesian approach which is based on the posterior distribution. The posterior distribution is proportional to the product of the prior and likelihood. We make use of the Empirical Bayes approach, which uses a hierarchical structure to determine the prior, where the prior is determined by a distribution with parameters called hyper-priors. The hyper-priors are estimated from the dataset which means that it is less affected by mis-specification of priors.

We also use the conventional Bayesian approach. For the conventional Bayesian approach, we set the priors of our *β*s to follow the normal distribution. For example, we set the prior of $\beta_{0}^{\left(1\right)}∼N$($\beta_{0}^{\left(1\right)};2,1$), γ∼β(γ;10,5) and set $\pi_{0}=0.6$. See Section 3 and Supplementary Material for more analysis on the sensitivity of using different priors.

The noise and signal components are allocated based on the prior information introduced into our prior distributions. For the two-component model, we considered that the smallest mean represents the noise component and the largest mean represents the mean signal. For the three-component model, we considered that the smallest mean represents the noise component, the intermediate mean represents the true signal and the largest mean represents the false signal. Thus, we labelled the first component as noise, the second component as true signal and the third component as false signal.

### 2.2 Datasets and preprocessing

#### 2.2.1 Drosophila dataset

To test the performance of the model, we used a high-resolution Hi-C map of Kc167 cell lines in *Drosophila* from Eagen *et al.* (2017). The raw data was downloaded and preprocessed with HiCExplorer following the set of parameters from Chathoth *et al.* (2022) and Chathoth and Zabet (2019). Briefly, we aligned each pair of the PE reads to *Drosophila melanogaster* (dm6) genome (dos Santos *et al.*, 2015) using BWA-mem (Li and Durbin, 2010) (with options -t 20 -A1 -B4 -E50 -L0). HiCExplorer was used to build and correct the contact matrices and detect enriched contacts (Ramirez *et al.*, 2018). The contact matrices were built using 2 Kb bins and then exported in text format to be loaded into R.

For DNA accessibility in *Drosophila* Kc167 cells data we used DNaseI-seq data from Kharchenko *et al.* (2011), while, for TE annotation in *Drosophila*, we used FlyBase (dos Santos *et al.*, 2015).

We detected TADs using HiCExplorer at 2 Kb resolution, similarly as done in Chathoth *et al.* (2022) and Chathoth and Zabet (2019). Briefly, TADs had at least 20 Kb width, a *P*-value threshold of 0.01, a minimum threshold of the difference between the TAD-separation score of 0.04, and FDR correction for multiple testing (–step 2000 –minBoundaryDistance 20 000 –pvalue 0.01 –delta 0.04—correctForMultipleTesting fdr).

#### 2.2.2 Human datasets

We also used Hi-C and micro-C datasets in H1-hES cells from Krietenstein *et al.* (2020). We used the same preprocessing pipeline as for the *Drosophila* dataset. Briefly, we aligned each pair to the human genome hg38 (Schneider *et al.*, 2017) using BWA-mem (Li and Durbin, 2010). HiCExplorer was used to build and correct the contact matrices at 10 Kb resolution and detect enriched contacts (Ramirez *et al.*, 2018).

Furthermore, we used DNaseI-seq for DNA accessibility from ENCODE consortium (Thurman *et al.*, 2012) and TE annotation from RepeatMasker http://www.repeatmasker.org.

### 2.3 Comparison to other tools

In this article, we compare our new method ZipHiC to three other tools: (i) FastHiC (Xu *et al.*, 2016a), (ii) HiCExplorer (Ramirez *et al.*, 2018) and (iii) Juicer (Durand *et al.*, 2016). First, we generate the enriched interactions using a JAVA implementation of FastHiC which uses expected counts and, for that, we used the values estimated by the HiCExplorer (Ramirez *et al.*, 2018).

Second, we used the HiCExplorer generated matrices and corrected them using the following values: (i) [-1.8,5.0] for Hi-C in Kc167 cells, (ii) [-2.4,5.0] for Hi-C in H1-hES cells, (iii) [-2.0,5.0] for micro-C in H1-hES cells, (iv) [-1.7,5.0] for Hi-C biological replicate 1 in Kc167 cells and (v) [-1.7,5.0] for Hi-C biological replicate 2 in Kc167 cells; see Supplementary Figure S1 (Ramirez *et al.*, 2018). Then, we generated the enriched contacts from the corrected matrix using hicFindEnrichedContacts tool with observed over expected method (--method obs/exp) (Ramirez *et al.*, 2018).

Third, we used Juicer to generate enriched contacts by calling dump tool from Juicer tools. In particular, we used the observed over expected method (oe) and Knight-Ruiz normalization (KR) at 2 Kb resolution for the Hi-C data in Kc167 cells and at at 10 Kb resolution for the Hi-C and micro-C data in H1-hES cells (Durand *et al.*, 2016).

Note that, to capture TE biases, we recommend not to use masking of the genome or to remove reads with multiple alignments (using –non-deterministic option if available).

The R scripts used to perform the analysis can be downloaded from https://github.com/igosungithub/HMRFHiC.git.

## 3 Results

### 3.1 Using the two-component model on simulated data

First, we considered the case of a two-component model (signal and noise) and evaluated whether this model can correctly estimate the sources of biases associated with Hi-C contact matrix using simulated data. We simulated a dataset of *n *=* *2500 observations from the mixture model (1), with *K *=* *2. The simulation studies are based on outputs of MCMC algorithms with 20 000 iterations and 10 000 burn-in steps. We considered using either informative prior or Empirical Bayes method, which has been used previously to analyze missing data (Carlin and Louis, 2000). Furthermore, there are three cases under different component proportions: (i) when the proportion of the noise is greater than the signal, (ii) when the proportion of the noise and the signal is the same, (iii) when the proportion of noise is less than the signal. Finally, we also used different starting values to justify the convergence of MCMC algorithms.

We studied the sensitivity of our model to different sets of prior parameters values using the traditional informative prior and Empirical Bayes method. The latter, the prior of the Empirical Bayes method, is based on the hyper-prior determined by the dataset. Supplementary Table S1 shows that the two-component model is able to estimate the true value accurately when using either the informative (fixed) or the Empirical Bayes method for the prior distribution. In order to illustrate the effect of using one of the priors (fixed prior or Empirical Bayes), we included only one covariate, *D_ij_* (genomic distance) from Equation (2). Our results show that the estimates of the posterior means of the parameters are accurate for both approaches of inferring the prior distribution. For our downstream analysis, we used the Empirical Bayes method.

Next, we evaluated the estimated posterior means of the parameters for our regression model (see Equation 2). We used a fixed informative prior and the component percentages (*α*s) in Equation (1) are set as $\alpha_{1}=0.7$ and $\alpha_{2}=0.3$, showing a higher percentage of noise to signal. Supplementary Table S2 shows that our method was able to estimate the true parameters accurately despite the higher noise. We also check our estimated posterior means with respect to their credible intervals, which are usually used in Bayesian analysis and have similar interpretation to confidence intervals. The main differences between our estimated posterior means and the true values we selected for our parameters fall within ±0.02, and our estimated posterior means are all significant as they fall within the 95% credible intervals. In addition, when evaluating Supplementary Tables S1 and S2 and analyzing the trace plots of all our simulations, we did not observe label switching; i.e. we are able to identify each components parameters distinctly without any unidentifiability issues. Furthermore, in Supplementary Tables S3 and S4, we show that our method is also robust to different proportions of noise and signal (see Supplementary Material).

### 3.2 Hi-C data analysis with a two-component model

Following the validation of our model on simulated data, we next used the two-component ZipHiC model on real Hi-C data. In particular, we used a dataset from Eagen *et al.* (2017) in a Kc167 cell line in *Drosophila* at 2 Kb resolution and focused our analysis on chromosome 2L. As mentioned earlier, the aim of our proposed method is to detect significant interactions, which we called true signal, by taking into consideration the biases associated with Hi-C dataset.

First, we considered the 31 375 observations from a 500 Kb region (2L:1–500 000), resulting in 250 unique pair of bins in order to compare our method to existing statistical methods. FastHic (Xu *et al.*, 2016a) is an updated version of the HMRFBayesHiC (Xu *et al.*, 2016b) as both methods use a hidden Markov random field (HMRF)-based Bayesian method and Ising model (Ising, 1925), which accounts for the spatial dependence in peak calling. Note that, we only used 31 375 observations, because of the high computation time of the FastHic (Xu *et al.*, 2016a). In contrast to ZipHiC, FastHic (Xu *et al.*, 2016a) method involves calculating the expected frequencies, which is computationally intensive and can be done using the approach in Lieberman-Aiden *et al.* (2009).

**Fig. 1.**
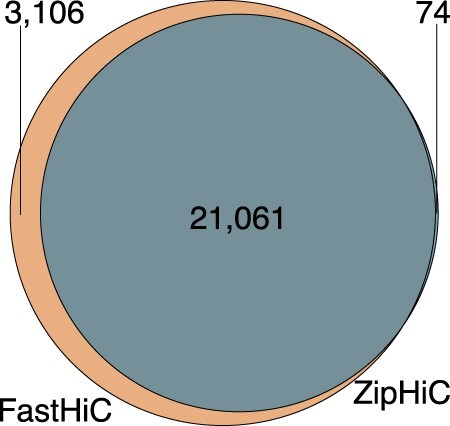
Comparison between ZipHiC and FastHiC Venn Diagram showing true signal comparison between our proposed method (ZipHiC) and FastHiC on sub region of chromosome 2L in *Drosophila* Kc167 cells. We considered that two interactions detected by the different tools are common if both anchors overlap fully, that is, the start and end of an anchor in one pair matches the start and end of corresponding anchor in the other pair. The parameters for detecting the significant interactions can be found in the Section 2

Based on the Monte Carlo draws from the posterior distribution of our ZipHiC model, we computed whether the estimated values of our parameters are significant or not (see posterior means values in Supplementary Tables S5 and S6 in Supplementary Material). Figure 1 shows the Venn diagram of the biologically significant interacting pairs of bins using ZipHiC two-component model compared to FastHic (Xu *et al.*, 2016a). ZipHiC recovers 87% (21 061) of the interactions detected by FastHic (Xu *et al.*, 2016a); see Figure 1. We noticed that the FastHic (Xu *et al.*, 2016a) method discovered an additional 3106 interactions as being biologically significant, suggesting that our model is slightly more conservative in detecting significant interactions. Interestingly, both methods detected 7134 interactions as noise (random collision). A further investigation of the additional significant interactions detected by the FastHic (Xu *et al.*, 2016a) and not by our method, showed that the FastHic (Xu *et al.*, 2016a) has a higher false discovery rate than our method by falsely classifying the interactions with 0 frequency as being significant.

### 3.3 Hi-C data analysis with a three-component model

One limitation of previous studies was the restriction to two components (noise and signal). Here, we further increased the number of components from *K *=* *2 to *K *=* *3 by adding a new component and we applied this model to the same 500 Kb region of chromosome 2L (2L:1–500 000). This new component accounts for interactions that ZipHiC has misclassified as signal due to conflicting information both in the contact frequencies and sources of bias and, thus, we call this new component *false signal*. For example, if a pair of interacting bins have high contact frequency (i.e. Hi-C retrieves a high number of interactions between the two regions of the genome), but their sources of bias closely exhibit that of the noise component, this pair of bins can be classified to the false signal component.

**Fig. 2.**
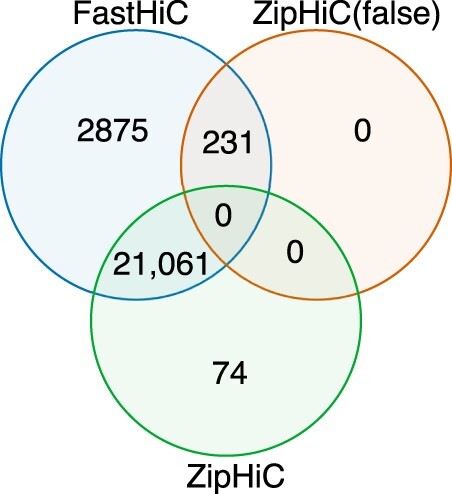
Venn Diagram showing comparison between the HMRF (Xu *et al.*, 2016b), ZipHiC-2 (our true signal) and ZipHiC-3 (our false signal) of the sub region of Chromosome 2L of *Drosophila Melanogaster*. We considered that two interactions detected by the different tools are common if both anchors overlap fully

First, we compared the detected significant interactions in the three-component ZipHiC model with the ones in the two-component one and from FastHic. Figure 2 shows that by adding an additional component, we detect less than 1% of additional interactions (231) overlapping with the FastHic (Xu *et al.*, 2016a) method.

To evaluate whether the new component in our method (false signal) results in better performance of our method, we conducted model selection analysis using the Deviance Information Criterion (Spiegelhalter *et al.*, 2002) and in particular, we used a modified DIC method (Li *et al.*, 2020) for latent variable models. The value of the DIC for the two-component model is -331 334 746 and for the three-component model is -401 662 547. These results show that the best model to analyze this particular Hi-C dataset is the three-component model (thus, including the false signal).

To better understand the contributions of the different components, we investigated the posterior means of our estimated *β*s for the noise, signal and false signal components (see Table 1). The values of *β*s correspond to the coefficients of the intercept and the log of genomic distance, GC content, TEs content and DNA accessibility. The posterior means of noise levels of the interaction for all components, except GC content, had *β* values with negative signs, indicating that the noise and signal were negatively correlated. The negative sign of *β*_1_ parameter (genomic distance) indicates that when genomic distance between two bins increases, then the average of their interaction noise decreases. Similarly, for *β*_3_ (TEs) and *β*_4_ (DNA accessibility), our results indicate that the higher the TEs content or the level of DNA accessibility is, then the lower the interaction noise will be, but only for DNA accessibility the effect is large. In other words, noise levels in the Hi-C signals are higher in dense chromatin and will have a higher impact on the observed enriched interactions, unless correctly accounted for. Nevertheless, for *β*_2_ (GC content), we found that higher GC content corresponds to a higher interaction noise. While this is significant, the contribution of GC content is relatively small to the noise levels in Hi-C data. Interestingly, we noticed in Supplementary Table S5 and Table 1 that our estimated posterior means for the noise components are similar if we use a two-component or a three-component model. This can be explained by the fact that most of the third component (false signal) in our model is influenced by the second component (true signal).

**Table 1.** Posterior means of our estimated *β*s as shown in Equation 2 for noise, signal and false signal components

Parameters	Posterior mean (noise), *k* = 1	Posterior mean (signal), *k* = 2	Posterior mean (false signal), *k* = 3
*β*_0_ (intercept)	−84.00 (-84.90, -83.64)	13.06 (12.80, 13.35)	499.34 (498.39, 500.21)
*β*_1_ (genomic distance)	−10.05 (−10.16, −10.03)	−0.90 (−0.92, −0.89)	−64.16 (−64.45, −63.97)
*β*_2_ (GC content)	0.34 (0.34, 0.35)	0.36 (0.35, 0.37)	0.30 (0.09, 0.57)
*β*_3_ (TEs)	−0.76 (−0.79, −0.68)	−0.10 (−0.16, −0.03)	0.54 (−0.40, 1.03)
*β*_4_ (Accessibility)	−3.54 (−3.57, −3.44)	0.15 (0.11, 0.20)	−0.70 (−1.04, −0.15)

*Note*: The 95% credible intervals are shown inside the brackets. The first component (*k *=* *1) represents the noise component, the second component (*k *=* *2) represents the signal component while the third component (*k *=* *3) represents the false signal component.

For the false signal component, we noticed that the posterior mean and credible intervals for the genomic distance (*β*_1_) parameter of the false signal component is significant. Furthermore, the negative value indicates that the increase in genomic distance of two bins results in a decrease in the false signal interaction. The effect size of genomic distance on false signal is higher than compared to noise and was previously unaccounted for. For DNA accessibility (*β*_4_), the negative value of the posterior mean and the credible intervals means that an increase in DNA accessibility leads to a decrease in the false signal interaction, but this is relatively small. Similarly for the posterior mean of the GC content (*β*_2_), the value is positive and indicates that higher GC content corresponds to an increase in the false signal. However for TEs (*β*_3_) the credible intervals of false signal component covers 0, which means the result is not significant.

Furthermore, we noticed that the posterior mean of true signal for GC content (*β*_2_) decreased when the third component (false signal) was added (compare from Tables 1 and Supplementary Table S5). This means that the influence of GC content was reduced when taking into account false signal. In addition, we noticed that the estimated posterior mean of (TEs) *β*_3_ for the signal component is significant and the false signal component is insignificant when the third component was added. This indicates that in order to properly estimate the true signal over TEs a three-component model might be required and previous models that did not include a false signal might have obtained inaccurate enriched contacts over TEs.

When we removed all the sources of bias (modelled as covariates in the regression model, equation 2), our method failed to detect any significant interactions in all possible 31 375 interactions from a 500 Kb region of the 2L chromosome (2L:1–500 000). The result clearly shows that the biases in the Hi-C data does affect the detection of significant interactions.

### 3.4 Whole chromosome analysis using the three-component ZipHiC model

**Fig. 3.**
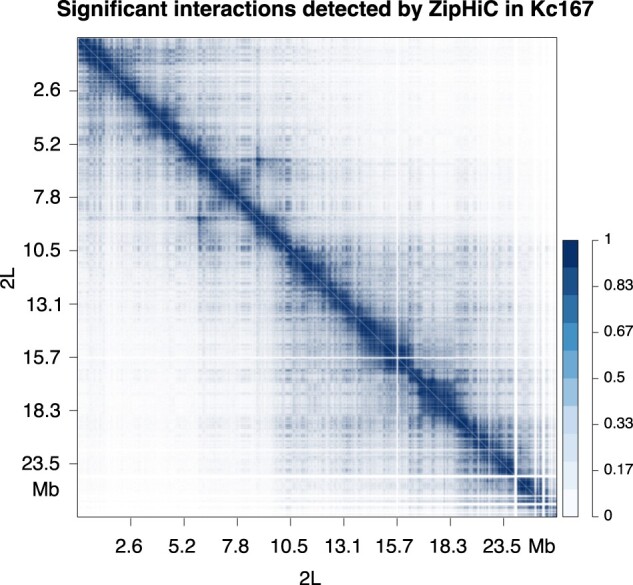
Significant interactions on chromosome 2L in Drosophila Kc167 cells. Heatmap showing significant interactions on chromosome 2L of *Drosophila* Kc167 cell line using ZipHiC three-component model. The intensity of the colour indicates the probability, with darker colours representing higher probability

Given that our model performs best with three components on this particular Hi-C dataset in *Drosophila* Kc167 cells, we analyzed the whole chromosome 2L (2L:1–23 513 700) using the three-component ZipHiC model and identified 12.82M significant interactions (see Supplementary Table S7 for the posterior means of the model). We observe that most of the detected significant interactions are found closer to the diagonal and that the significant interactions formed triangular shapes along the diagonal which sometimes overlap each others; see Figure 3.

**Fig. 4.**
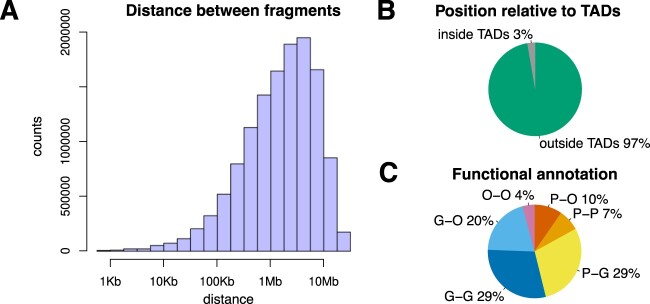
Characterization of significant interactions on chromosome 2L in Drosophila Kc167 cells. (**A**) Distribution of the genomic distance between the two bins for all significant interactions. (**B**) Classification of significant interactions as either outside TADs when the two bins are located in different TADs or inside TADs when the two bins are located in the same TAD. (**C**) Percentage of significant interactions that have promoters at one of the bins. We consider the cases of: (P) promoters (200 bp upstream and 50 bp downstream of TSS), (G) genes (including exons, introns, 5’UTRs and 3’ UTRs and excluding promoters) and (O) other regions (excluding promoters and genes)

These triangular shapes resemble Topologically Associated Domains (TADs) (Dixon *et al.*, 2012; Hansen *et al.*, 2018; Nora *et al.*, 2012; Sexton *et al.*, 2012) and are one of the main features of Hi-C data. However, we found that the majority of significant interactions connect regions of the genome that are very far apart (between 1 Mb and 10 Mb) (see Fig. 4A), which are genomic distances larger than the usual size of TADs in *Drosophila* (Chathoth *et al.*, 2022; Chathoth and Zabet, 2019; Ramirez *et al.*, 2018) and suggests that they connect bins located in different TADs. Indeed, this is the case and approximately 98% of significant interactions are outside TADs (see Fig. 4B). Interestingly, we found that almost half of the significant interactions connect promoters with other parts of the genome or with other promoters, which indicates they have a functional role (see Fig. 4C). The majority of the significant interactions connect genes with either themselves or other genes, promoters or other regions of the genome (potentially enhancers). Note that we also performed a genome wide analysis and these results are true for all chromosomes (see Supplementary Fig. S2).

**Fig. 5.**
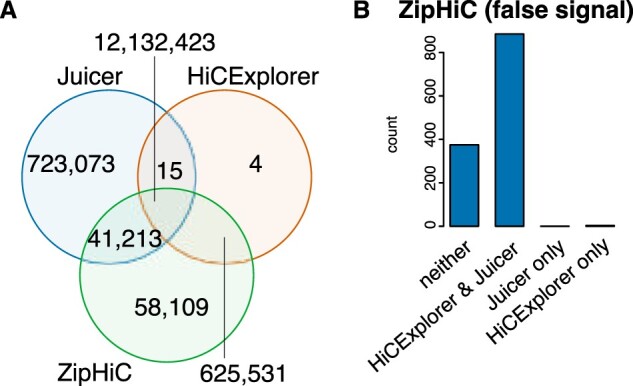
Comparison with other tools. (**A**) Venn Diagram showing the comparison between significant interactions detected by ZipHiC, HiCExplorer and Juicer. We analyzed chromosome 2L in *Drosophila* Kc167 cells. We considered that two interactions detected by the different tools are common if both anchors overlap fully. (**B**) The number of false signals identified by ZipHiC detected as true signals by HiCExplorer and Juicer

Finally, we compared the significant interaction detected by ZipHiC with significant interactions detected by two popular tools: HiCExplorer (Ramirez *et al.*, 2018) and Juicer (Durand *et al.*, 2016). Figure 5A shows that high proportions of significant interactions detected by ZipHiC are common with both HiCExplorer and Juicer (12.1M). In addition, ZipHiC detects 625K interactions detected only by HiCExplorer and missed by Juicer and 41K significant interactions detected only by Juicer and missed by HiCExplorer. ZipHiC uniquely identifies 58K significant interactions, which are missed by the other tools. Overall, we found that ZipHiC recovers almost all HiCExplorer (12.75M) significant interactions (99.2% overlap), but also an additional 99K significant interactions missed by HiCExplorer. Significant interactions detected by Juicer have a smaller overlap with the ones identified by ZipHiC (94.6%), but Juicer also retrieves approximately 723K unique significant interactions. Also in Figure 5A, we noticed that 15 significant interactions detected both by Juicer and HiCExplorer were missed by the ZipHiC.

Figure 5B shows the overlap between the interactions classified as false signal by ZipHiC and the significant interactions detected by the other methods (HiCExplorer and Juicer). ZipHiC detected 1263 significant interactions on chromosome 2L as false signal. 885 of these were detected as significant interactions by both Juicer and HiCExplorer, further supporting the fact that these tools are affected by false signal. Nevertheless, 375 interactions that were detected as false signal by ZipHiC were correctly not identified by HiCExplorer and Juicer as significant interactions, indicating that these tools can correctly remove some artefacts from the Hi-C data.

Finally, we evaluated the robustness of the identified significant interactions by running ZipHiC on chromosome 2L for two independent biological replicates. We identified approximately 8.3M significant interactions and observed an overlap between the two biological replicates of approximately 47% (see Supplementary Fig. S3). We further investigated the posterior means of the models of the two replicates and found that there are negligible differences between the two replicates except for two components (Supplementary Table S8). In particular, replicate 1 shows a high posterior mean for false signal for the TEs component (4.4), which indicates that higher TE content results in higher false signal interactions. In addition, we also found that replicate 1 displays a high negative posterior mean for false signal for the accessibility component (-5.1) indicating that dense chromatin leads to higher false signal interactions. Altogether, our results indicate that replicate 1 might be affected by a higher level of false positive significant interactions at regions with high TE content and dense chromatin.

This overlap between the two biological replicates is consistent with the overlap of significant interactions between the two replicates when using HiCExplorer and Juicer (see Supplementary Fig. S3) and can be explained by the lower library sizes. After pre-processing, replicate 1 had 239M valid interactions and replicate 2 had 247M valid interactions. That is approximately half of the merged library, which had 474M valid interactions. Lower library sizes result in more zeros in the interaction matrix and lead to less reliable detection of significant interactions. Instead of merging biological replicates, one alternative approach consists of selecting the overlap of significant interactions between biological replicates, similar to ENCODE recommendations for ChIP-seq data analysis (Landt *et al.*, 2012). This will ensure selection of a high confidence set of significant interactions, but at the same time would result in missing some significant interactions.

### 3.5 Analysis of micro-C data in human ES cells

Micro-C is a new and improved variation of Hi-C that can generate sub-kilobasepair 3D contacts map in mammalian systems (Hsieh *et al.*, 2015; Krietenstein *et al.*, 2020). To evaluate the capacity of ZipHiC to analyze micro-C data, we consider a small region on human chromosome 8 (60-70 Mb) for which both micro-C and Hi-C data is available in human ES cells (Krietenstein *et al.*, 2020). As we did previously, we consider both a two-component and a three-component model (*K *=* *2 and *K *=* *3) and use the DIC to select the best performing model (for the 3 components models of Hi-C and micro-C data see Supplementary Tables S9 and S10, respectively). Interestingly, in the case of this specific region on the human chromosome 8, the two-component model has the lowest DIC (${\text{DIC}}_{2}=194\;721.1$ and ${\text{DIC}}_{3}=469\;950.5$) and, thus, was selected for the analysis. This indicates that the human ES cell Hi-C and micro-C data in this region of the genome is not affected by false positive signals as it was the case with the *Drosophila* whole genome analysis in Kc167 cells.

**Fig. 6.**
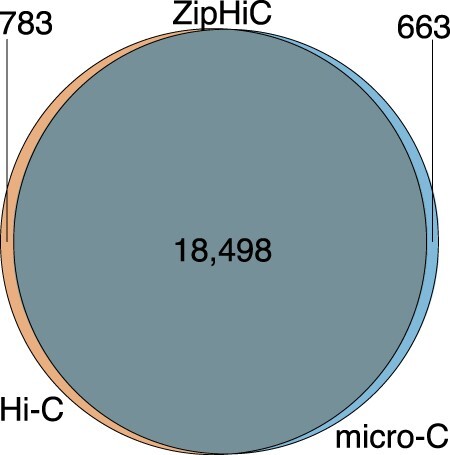
Venn Diagram showing significant interactions (signal) comparison identified by ZipHiC on micro-C and Hi-C data in human ES cells within 60–70 Mb region of human chromosome 8. We considered that two interactions detected by the different tools are common if both anchors overlap fully. The parameters for detecting the significant interactions can be found in the Section 2

Figure 6 shows that 96% (18 498) of significant interactions identified by ZipHiC in the Hi-C dataset are recovered as significant interactions in the micro-C dataset for this particular region of the human genome (60-70 Mb) and only a negligible number of interactions are missed (4%). Similarly, only 3% of the micro-C interactions are novel and previously missed by Hi-C. Our results confirm that micro-C can reproduce accurately the results of Hi-C despite a significantly lower library size.

We also investigated the overlap between the significant interactions identified by ZipHiC, Juicer and HiCExplorer and found that the three methods agree well (see Supplementary Fig. S3). Nevertheless, ZipHiC was also able to analyze the models and extract the sources of bias in the Hi-C and micro-C datasets. In micro-C, the chromatin is fragmented to mononucleosomes using micrococcal nuclease (MNase), which increases fragment density. The digestion with MNase raises the possibility that micro-C data is affected by DNA accessibility biases, which would not be the case with Hi-C data.

Table 2 shows the model parameters for the two-component model for both micro-C and Hi-C data. Interestingly, we observe that the effect of DNA accessibility on the mean signal is higher even compared to the effect of the genomic distance between the bins on the mean signal. A similar effect in the mean signal was also observed in the case of Hi-C data, but that was approximately half compared to the level observed in the micro-C data. In the case of the whole genome Hi-C analysis in *Drosophila*, we identified limited effects of accessibility on the mean signal but strong effects on the noise component. For this particular region in the human genome, we observed the opposite, strong biases introduced by accessibility in the mean signal (especially in the micro-C data), but significantly reduced biases on the noise component. The beta values have a positive sign indicating that more accessible regions of the genome display a higher signal, but only modest biases in the noise levels.

**Table 2.** Posterior means of our estimated *β*s as shown in Equation 2 for noise and signal components of human Chromosome 8, region 60 000 000:70 000 000 for data generated using the Hi-C and micro-C method

Parameters (Hi-C)	Posterior mean (noise), *k* = 1	Posterior mean (signal), *k* = 2
*β*_0_ (intercept)	0.88 (0.53, 1.36)	11.13 (10.92, 11.36)
*β*_1_ (genomic distance)	0.13 (−0.02, 0.25)	−0.79 (−0.81, −0.77)
*β*_2_ (GC content)	0.33 (0.32, 0.33)	0.32 (0.32, 0.34)
*β*_3_ (TEs)	1.01 (0.99, 1.15)	0.02 (0.01, 0.03)
*β*_4_ (Accessibility)	0.50 (0.43, 0.59)	1.00 (0.99, 1.03)

Parameters (micro-C)	Posterior mean (noise), *k* = 1	Posterior mean (signal), *k* = 2

*β*_0_ (intercept)	1.05 (0.81, 1.30)	8.08 (7.65, 8.39)
*β*_1_ (genomic distance)	0.14 (0.12, 0.17)	−1.41 (−1.42, −1.38)
*β*_2_ (GC content)	0.33 (0.32, 0.34)	1.02 (0.12, 1.80)
*β*_3_ (TEs)	10.00 (9.99, 10.02)	−0.37 (−0.41, −0.33)
*β*_4_ (Accessibility)	0.40 (0.35, 0.41)	1.83 (1.70, 1.92)

*Note*: The 95% credible intervals are shown inside the brackets. The first component (*k *=* *1) represents the noise component, the second component (*k *=* *2) represents the signal component.

Furthermore, we also identified a strong contribution to the noise of the signal from the TE content. This was particularly in the micro-C dataset, but also present in the Hi-C data despite being ten times lower. This means that a higher TE content leads to a higher noise, specifically in the micro-C data. In addition, micro-C data also display low bias of TE content in the mean signal, indicating that higher TE content leads to a slightly lower signal in micro-C, but not in Hi-C. Note that in the case of whole genome analysis in *Drosophila*, there was only a relatively medium bias from TE content in the noise and false signal components, but not in the true signal component.

## 4 Discussion

In this manuscript, we introduce a new method called ZipHiC to analyze Hi-C and micro-C data. ZipHiC models the contact frequencies as a Zero-Inflated Poisson distribution due to the fact that this enables modelling the presence of the overdispersion which affects Hi-C data (Varoquaux *et al.*, 2021). In addition, ZipHiC also uses a hidden Markov Random Field (HMRF)-based Bayesian method, the Potts model, to help account for dependency in Hi-C dataset. Most importantly, the Potts model allows an increase in the number of components (k=2,3,…K) and, thus, to account for additional components such as false signal. Finally, our method uses a likelihood free approach, ABC, to account for the limitation in the normalizing constant in the Potts model. Through our extensive simulations on simulated and real data, we show that our method outperforms existing methods in distinguishing between noise and signal.

First, we found that a three-component model (specifically considering the false signal) performed better on a very high resolution dataset in *Drosophila* Kc167 cells (Eagen *et al.*, 2017). However, a two-component model (considering only the noise and the signal) performed best for the Hi-C and micro-C datasets in human ES cells (Krietenstein *et al.*, 2020) on a region on chromosome 8. This indicates that the choice of whether to use a two-component or a three-component model needs to be driven by the data, since not all datasets will be affected by a false signal(s) component. In addition, we identified different biases between different organisms (*Drosophila* and *humans*) that are affected by different TE composition or DNA accessibility, but also between different techniques on the same material. This indicates that there are sample specific biases that can affect the identification of significant interactions.

In *Drosophila*, we found that the genomic distance between bins has the highest contribution to both the noise and the false signal, where interactions further from the diagonal display less noise and fewer false signals compared to interactions closer to the diagonal. DNA accessibility contributed strongly to the noise component and partially to the false signal in *Drosophila*. In particular, less accessible regions of the genome displayed higher noise and more false signals. We also observed a moderate effect of TEs on the noise component and false signal in *Drosophila*, where regions with higher content of TEs displayed lower noise, but higher false signals.

The majority of these significant interactions connect regions of the genome that are located in different TADs and this is explained by the larger genomic distance between the two bins detected by ZipHiC in this dataset. The genomic distance between bins is larger than previously reported in *Drosophila* cells (Chathoth and Zabet, 2019), due to the fact that in this study we used a 2 Kb resolution and in the previous study a higher resolution was used (DpnII restriction sites, on average every 529 bp).

Most importantly, we identified that approximately half of these significant interactions in *Drosophila* connect promoters with either other promoters, genes or other regions of the genome. This raises the possibility that these significant interactions connect promoters with regulatory regions. Nevertheless, the large number of detected significant interactions and the number of enhancers identified in *Drosophila* cells (Arnold *et al.*, 2013; Wolfe *et al.*, 2021; Yanez-Cuna *et al.*, 2014), indicate that most of them would not connect promoters with enhancers. This is likely the case and one possibility is that a large part of the significant interactions account for gene domains being formed at actively transcribed genes, where the promoter of the gene makes 3D contacts with different parts of the gene (exons, introns or 3’UTRs) (Rowley *et al.*, 2019). Indeed, we found that the majority of significant interactions involve genes, further supporting this model.

Furthermore, we found that micro-C data reproduces the majority of the significant interactions (96%) detected on a much larger Hi-C library. However, the micro-C data displays a higher bias in the signal to DNA accessibility (more accessible regions of the genome will display higher signals) even compared to genomic distance between the bins and this needs to be accounted for. Interestingly, in this particular region, the noise component was particularly affected by the TE content, where more TEs lead to a higher noise in the micro-C data. The stronger effect of TEs on micro-C data in human cells is not surprising given the fact that human genome has a higher percentage of TEs compared to *Drosophila*.

Our model uses the DNA accessibility, TE content and GC content as external inputs to compute the biases introduced by these factors when detecting significant interactions from HiC data. One question that arises is whether accessibility, TE content and GC content are truly experimental biases or factors contributing to the 3D genome organization. One would expect that if these factors (TE content, accessibility and GC content) would impact the 3D genome architecture and are not introducing biases in the experiments, then their relative contribution would be the same in different experiments on the same material. For example, when performing the Hi-C and micro-C on the same material, we expect that accessibility has the same posterior mean of the true signal for both experiments. However, what our results show is that in the case of micro-C the value is almost double as in the case of Hi-C. This suggests that it is not the underlying biology driving this, but, most likely, these are experimental biases. Nevertheless, our work cannot exclude that accessibility, TE content and GC content have some contribution to the 3D genome organization. For example, TEs have the possibility to move binding sites for architectural proteins throughout the genome (Schmidt *et al.*, 2012) and, in this scenario, presence of TEs would contribute to the observed 3D chromatin organization. However, aligning reads from genomics libraries (including Hi-C) to regions of the genome containing TEs is often challenging and, thus, high TE content would correspond to higher biases in the HiC data (Taylor *et al.*, 2022).

A limitation of ZipHiC compared to tools such as HiCExplorer and Juicer is the computation time when analyzing whole genomes. In the case of a standard computer with 4 cores, ZipHiC takes approximately 72 hours to analyze a whole genome dataset in *Drosophila* at 2 Kb resolution. This is slower compared to HiCExplorer and Juicer, which can detect the significant interactions for the same dataset in approximately 4 hours on a similar computer system. Note however that, ZipHiC models additional features compared to HiCExplorer and Juicer, namely it models spatial information and allows multiple components. Compared to another tool that models spatial information and only two components (FastHiC), ZipHiC is faster; i.e. we were not able to run FastHiC on whole chromosome 2L in *Drosophila* at 2 Kb resolution within a feasible time.

## Supplementary Material

btac387_Supplementary_Data
