# Supplementary material for: ZipHiC: a novel Bayesian framework to identify enriched interactions and experimental biases in Hi-C data
Source: Bioinformatics. 2022 Jun 9;38(14):3523–31. doi: 10.1093/bioinformatics/btac387 (PMC9272800; doi:10.1093/bioinformatics/btac387)
Supplement: btac387_Supplementary_Data [file btac387_supplementary_data.pdf]

Supplementary Material to *ZipHiC: a novel Bayesian framework to identify enriched interactions and experimental biases in Hi-C data*

S1 Supplementary Figures

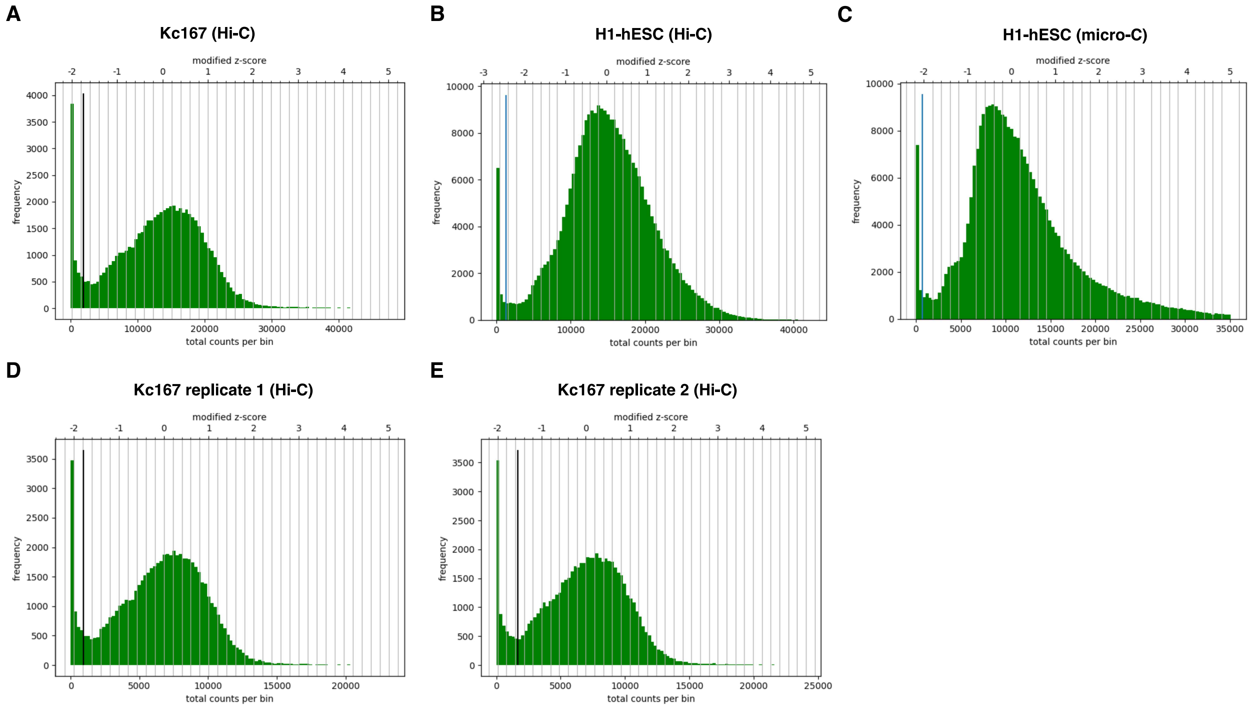

Figure S1: *Diagnostic plots for correction of Hi-C plots from HiCExplorer.* Histograms of the sum of contacts per bin. The vertical black line represents the lower threshold for removing bins with lower number of reads. We plotted the histograms for the three datasets used in this study: (A) Hi-C in Kc167, (B) Hi-C in H1-hES cells, (C) micro-C in H1-hES cells, (D) Hi-C replicate 1 in Kc167 and (E) Hi-C replicate 1 in Kc167.

S2 Model for Data

The complete likelihood function of the unknown parameters $(\boldsymbol{\beta}, \boldsymbol{\alpha}, \boldsymbol{\tau})$ given the data y given z can then be written as

$$l(\vec{\boldsymbol{\beta}}, \vec{\boldsymbol{\alpha}}, \boldsymbol{\tau}|\boldsymbol{y}, \boldsymbol{z}) \propto \prod_{i=1}^{n}\prod_{j=1}^{n}\left\{\left[\alpha_1\left(\tau + (1-\tau)e^{-\lambda_{ij}^{(1)}} + (1-\tau)\frac{\left(\lambda_{ij}^{(1)}\right)^{y_{ij}} e^{-\lambda_{ij}^{(1)}}}{y_{ij}!}\right)\right]^{I(z_{ij}=1)} \prod_{k=2}^{K}\left[(\alpha_k)\frac{\left(\lambda_{ij}^{(k)}\right)^{y_{ij}} \exp\left(-\lambda_{ij}^{(k)}\right)}{y_{ij}!}\right]^{I(z_{ij}=k)}\right\} \quad \text{(S1)}$$

The full posterior of z, $\vec{\boldsymbol{\beta}}$ and γ given y_{ij} is

$$Pr(\boldsymbol{z}, \vec{\boldsymbol{\beta}}, \gamma|\boldsymbol{y}) \propto l(\vec{\boldsymbol{\beta}}, \vec{\boldsymbol{\alpha}}, \boldsymbol{\tau}|\boldsymbol{y}, \boldsymbol{z})l(\boldsymbol{z}|\gamma)\pi_0(\gamma)\tau_0(\vec{\boldsymbol{\beta}}) \quad \text{(S2)}$$

where $l(\boldsymbol{z}|\gamma) = \frac{e^{\gamma\sum_{s\sim t}\delta_{z_s z_t}}}{\sum_{z_s} e^{\gamma\sum_{s\sim t}\delta_{z_s z_t}}}$ is the Potts model, s is bin pair i and j, and t is the neighbours set of s; $(i-1, i+1, j-1, j+1)$.

In order to analyse our data and estimate our parameters, we make use of the Metropolis-within-Gibbs sampler and the Approximate Bayesian Computation (ABC), so the conditional posterior densities are needed.

S2.1 Conditional Posterior Density

The conditional posterior of τ is given as

$$Pr(\tau|\vec{\boldsymbol{\beta}}^{\mathbf{1}}, \boldsymbol{y}, \boldsymbol{z}) \propto \prod_{i=1}^{n}\prod_{j=1}^{n}[f^{(1)}(y_{ij}; \boldsymbol{\beta}^{(1)})]^{I[z_{ij}=1]} \cdot \pi_0(\tau) \quad \text{(S3)}$$

The conditional posterior of $\boldsymbol{\beta}$ is given by

$$Pr(\vec{\boldsymbol{\beta}}^{(\boldsymbol{k})}|\boldsymbol{y}, \boldsymbol{z}) \propto \prod_{i=1}^{n}\prod_{j=1}^{n}\prod_{k}^{K}[f^{(k)}(y_{ij}; \boldsymbol{\beta}^{(k)})]^{I[z_{ij}=k]}\pi_0(\boldsymbol{\beta}^{(k)}) \quad \text{(S4)}$$

Based on the definitions of $f^{(k)}(y_{ij})$ and equation S4, the conditional posterior of $\boldsymbol{\beta}^{(1)}$ for the noise component can be rewritten as

$$Pr(\vec{\boldsymbol{\beta}}^{(\mathbf{1})}|\boldsymbol{y}, \boldsymbol{z}) \propto \prod_{i=1}^{n}\prod_{j=1}^{n}\left[\left(\tau + (1-\tau)e^{-\lambda_{ij}^{(1)}} + (1-\tau)\frac{\left(\lambda_{ij}^{(1)}\right)^{y_{ij}} e^{-\lambda_{ij}^{(1)}}}{y_{ij}!}\right)\right]^{I(z_{ij}=1)} \exp\left\{-\frac{(\beta^{(1)} - m^{(1)})^2}{2(\sigma^2)^{(1)}}\right\} \quad \text{(S5)}$$

For the signal component, the conditional posterior of $\boldsymbol{\beta^{(k)}}$ based on definition of $f^{(k)}(y_{ij})$ and equation S4 can be rewritten as

$$Pr(\vec{\boldsymbol{\beta}}^{\boldsymbol{(k)}}|\boldsymbol{y},\boldsymbol{z}) \propto \prod_{i=1}^{n}\prod_{j=1}^{n}\left[\frac{\left(\lambda_{ij}^{(k)}\right)^{y_{ij}}\exp\left(-\lambda_{ij}^{(k)}\right)}{y_{ij}!}\right]^{I(z_{ij}=k)} \exp\left\{-\frac{(\beta^{(k)}-m^{(k)})^2}{2(\sigma^2)^{(k)}}\right\} \quad \text{(S6)}$$

where $m^{(k)}$ is the mean and $\left(\sigma^{(k)}\right)^2$ is the variance for component k.

To update the latent variable, the probability of an observation belonging to each component is calculated

$$Pr(z_s|\gamma, z_t, \boldsymbol{y}, \vec{\boldsymbol{\beta}}^{\boldsymbol{(k)}}) \propto e^{\gamma\sum_{s\sim t}\delta_{z_s z_t}} f(y_{i,j};\boldsymbol{\beta}^{(k)}) \quad \text{(S7)}$$

where s is bin pair i and j, and t is the neighbours set of s; $(i-1,\ i+1,\ j-1,\ j+1)$ and $f^{(k)}(y_{ij};\vec{\boldsymbol{\beta}}^{\boldsymbol{(k)}})$ is the likelihood of component k.

When the normalizing constant is introduced, equation S7 can be rewritten as

$$Pr(z|\gamma, z_t, \boldsymbol{y}, \vec{\boldsymbol{\beta}}^{(k)}) = \frac{e^{\gamma\sum_{s\sim t}\delta_{z_s z_t}}}{\sum_{z_s} e^{\gamma\sum_{s\sim t}\delta_{z_s z_t}}}\frac{f(y_{i,j};\boldsymbol{\beta}^{(k)})}{f(y_{i,j};\boldsymbol{\beta}^{(k)})} \quad \text{(S8)}$$

where s is bin pair i and j, and t is the neighbour(s).

The conditional probability of γ in the Potts model is given as

$$Pr(\gamma|\boldsymbol{y},\boldsymbol{z},\vec{\boldsymbol{\beta}}) = \frac{\exp\{\gamma\sum_{s\sim t}\delta(z_s z_t)\}\pi_0(\gamma)}{\sum_{z_s}\exp\{\gamma\sum_{s\sim t}\delta(z_s z_t)\}\pi_0(\gamma)} \quad \text{(S9)}$$

Algorithm S1 ABC

```
procedure
  repeat
    Select the initial value of γ^0;
    m = 0
    for i = 1 : N do
      Compute a new y* based on the Potts model and updated β⃗ from Algorithm (5.2)
      Compute the distance d(S(y*), S(y))
      Select ϵ using 1% empirical quantile of d(S(y*), S(y))
      if d(S(y*), S(y)) < ϵ then
        γ_{m+1} = (γ_m)*
      end if
    end for
  until enough MCMC steps have been simulated;
```

Algorithm S2 Metropolis-within-Gibbs sampler

procedure
    Initialization, select initial value, $z^0, \gamma^0, \tau^0, \vec{\boldsymbol{\beta}}^0$;
    repeat
        for $i = 1$ to n, $j = 1$ to n **do**
            Update z_{ij} using (5.13)
        end for
        Update $\vec{\boldsymbol{\beta}}$ from posterior in (5.10)
        Update γ using Algorithm (S1)
        Update τ using (5.8)
    until enough MCMC steps have been simulated;

The equations for $\vec{\boldsymbol{\beta}}$'s and z as given above in equations S3, S4 and S7, is computationally easier to simulate using the Metropolis-Hastings-within-Gibbs sampler. Equation S9 is computationally intractable as the interaction parameter γ involves the evaluation of the partition function and cannot be simulated directly using the Gibbs sampler or the Metropolis-Hastings sampler.

Algorithm S1 shows the Approximate Bayesian Computation (ABC) approximate steps and Algorithm S2 shows the Metropolis-within-Gibbs steps used in this paper to update our parameters.

S2.2 Analysis

Table S1: Simulation results for normal priors. $\beta_0^{(1)}$ and $\beta_1^{(1)}$ are the Intercept and genomic Distance parameters of the noise component, while $\beta_0^{(2)}$ and $\beta_1^{(2)}$ are the Intercept and genomic Distance parameters of the signal components. In brackets we presented the 95% credible intervals. For the fixed prior, we used $\beta_0^{(1)} \sim N(1, 1)$, $\beta_0^{(2)} \sim N(150, 10)$, $\beta_1^{(1)} \sim N(0.5, 0.5)$ and $\beta_1^{(2)} \sim N(1, 0.5)$.

Parameters	True value	Posterior mean (fixed prior)	Posterior mean (empirical bayes method)
$\beta_0^{(1)}$	0.05	0.04 (0.01, 0.05)	0.04 (0.01, 0.07)
$\beta_1^{(1)}$	0.2	0.21 (0.24, 0.29)	0.22 (0.2, 0.27)
$\beta_0^{(2)}$	5.00	4.99 (4.99, 5.00)	5.00 (4.98, 5.01)
$\beta_1^{(2)}$	2	1.98 (1.99, 2.04)	2.02 (1.93, 2.08)

Table S2: Simulation results for normal priors. $\beta_0^{(1)}$, $\beta_1^{(1)}$, $\beta_2^{(1)}$,$\beta_3^{(1)}$ and $\beta_4^{(1)}$ are the Intercept, genomic Distance, GC content, TEs and Accessibility parameters of the noise component, while $\beta_0^{(2)}$, $\beta_1^{(2)}$, $\beta_2^{(2)}$ and $\beta_3^{(2)}$ are the Intercept, genomic Distance, GC content, TEs and Accessibility parameters of the signal components. In bracket are the 95% credible intervals.

Parameters	True value	Posterior mean
$\beta_0^{(1)}$ (intercept)	0.05	0.04 (0.05, 0.14)
$\beta_1^{(1)}$ (genomic distance)	0.2	0.18 (0.13, 0.24)
$\beta_2^{(1)}$ (GC content)	0.3	0.30 (0.24, 0.35)
$\beta_3^{(1)}$ (TEs)	0.2	0.19 (0.14, 0.25)
$\beta_4^{(1)}$ (Accessibility)	0.1	0.08 (0.02, 0.13)
$\beta_0^{(2)}$ (intercept)	5	5.01 (4.96, 5.06)
$\beta_1^{(2)}$ (genomic distance)	2	2.01 (1.96, 2.05)
$\beta_2^{(2)}$ (GC content)	0.8	0.80 (0.75, 0.84)
$\beta_3^{(2)}$ (TEs)	0.7	0.69 (0.65, 0.74)
$\beta_4^{(2)}$ (Accessibility)	0.6	0.60 (0.55, 0.65)

Table S3: Simulation results for normal priors when the proportion of signal = noise. $\beta_0^{(1)}$ and $\beta_1^{(1)}$ are the Intercept and genomic Distance parameters of the noise component, while $\beta_0^{(2)}$ and $\beta_1^{(2)}$ are the Intercept and genomic Distance parameters of the signal components. In bracket are the 95% credible intervals.

Parameters	True value	Posterior mean (empirical Bayes method)
$\beta_0^{(1)}$	0.05	0.06 (0.02, 0.11)
$\beta_1^{(1)}$	0.2	0.20 (0.15, 0.24)
$\beta_0^{(2)}$	5	5.00 (4.97, 5.03)
$\beta_1^{(2)}$	2	2.02 (1.83, 2.21)

As mentioned in the main text, we simulated only one source of bias (genomic distance) to see how our method performed to different proportions of noise and signal due to the computation time. Table S3 shows the result of the simulation study when the proportion of noise and signal are the same. In Table S3, when the proportion of noise and signal in the simulated data are the same, we can see that our method using the empirical Bayes method adequately estimated the true value of the parameters in our simulated data. Table S4 shows the result of the simulation study when the proportion of noise is less than that of signal proportion. We can see that our method using the empirical Bayes method as shown in Table S4 adequately estimated the true value of the parameters in our simulated data. The 95% credible intervals in both Tables S3 and S4 are all significant.

Table S4: Simulation results for normal priors when the proportion of noise = 0.3 and the proportion of signal = 0.7. $\beta_0^{(1)}$ and $\beta_1^{(1)}$ are the Intercept and genomic Distance parameters of the noise component, while $\beta_0^{(2)}$ and $\beta_1^{(2)}$ are the Intercept and genomic Distance parameters of the signal components. The 95% credible intervals are in the brackets.

Parameters	True value	Posterior mean (empirical Bayes method)
$\beta_0^{(1)}$	0.05	0.09 (0.03, 0.19)
$\beta_1^{(1)}$	0.2	0.16 (0.11, 0.20)
$\beta_0^{(2)}$	5	5.00 (4.98, 5.02)
$\beta_1^{(2)}$	2	1.96 (1.74, 2.21)

S2.3 Hi-C Data analysis with a two-component model

Table S5: Posterior means of our estimated βs for both noise and signal components. The 95% credible intervals are shown inside the brackets.

Parameters	Posterior mean (noise), k=1	Posterior mean (signal), k=2
β_0 (intercept)	-84.26 (-85.26, -83.29)	12.24 (11.26, 13.22)
β_1 (genomic distance)	-10.10 (-11.08, -9.12)	-0.92 (-1.90, -0.06)
β_2 (GC content)	0.35 (-0.64, 1.34)	4.38 (3.40, 5.37)
β_3 (TEs)	-0.73 (-1.73, 0.23)	-0.02 (-1.00, 0.96)
β_4 (Accessibility)	-3.51 (-4.49, -2.52)	0.07 (-0.91, 1.05)

From Table S5, we can see that the credible intervals for β_2 and β_3 (GC content and TEs) are not significant at 95% credible intervals for the noise component. For the signal component, β_3 and β_4 (TEs and Accessibility) are not significant. Due to about half of our covariates not being significant, when we set our credible interval as 95%, we instead use 90% for our analysis.

To better understand the contributions of the different components, we investigated the posterior means of our estimated βs for both the noise and signal components (see Table S6).

For the signal component, we noticed that the posterior means of the coefficient of the genomic distance and TEs (β_1 and β_3) are negative values. While the posterior means of the intercept, GC content and DNA accessibility (β_0, β_2 and β_4) are all positive values. In addition, the credible intervals for the genomic distances for all the components (β_1) are significant and the negative posterior mean indicates that as the genomic distance of two bins increases, the average of their signal interaction decreases as well. Similarly for the other significant parameters, β_2 (GC content) and β_4 (DNA accessibility), their positive values for posterior means and credible intervals indicates that as GC content and level of DNA accessibility increases the average of the signal interaction

Table S6: Posterior means of our estimated $\vec{\beta}$s for both noise and signal components. The 90% credible intervals are shown inside the brackets.

Parameters	Posterior mean (noise), k=1	Posterior mean (signal), k=2
β_0 (intercept)	-84.26 (-84.86, -83.73)	12.24 (11.86, 12.60)
β_1 (genomic distance)	-10.10 (-10.14, -10.06)	-0.92 (-0.94, -0.90)
β_2 (GC content)	0.35 (0.34, 0.35)	4.38 (3.71, 5.07)
β_3 (TEs)	-0.73 (-0.79, -0.70)	-0.02 (-0.07, 0.03)
β_4 (Accessibility)	-3.51 (-3.55, -3.46)	0.07 (0.02, 0.10)

increases as well. In other words, our results indicate that there is a small impact of DNA accessibility on the Hi-C results, where regions with higher DNA accessibility are retrieved more often than regions in dense chromatin, but this bias is small. However for β_3 of the signal component, the credible intervals is not significant as it is having 0 in-between. Altogether, our results show that genomic distance between pairs of loci and the GC content are the most significant sources of bias in our Hi-C data (Table S6).

S2.4 Genome wide analysis of *Drosophila* Kc167 cells

Table S7: Posterior means of our estimated $\vec{\beta}$s for noise, signal and false signal components for the whole chromosome 2L. The 95% credible intervals are shown inside the brackets.

Parameters	Posterior mean (noise), k=1	Posterior mean (signal), k=2	Posterior mean (false signal), k=3
β_0 (intercept)	-24.42 (-24.77, -23.92)	11.96 (11.68, 12.19)	498.82 (493.86, 503.73)
β_1 (genomic distance)	-2.63 (-2.68, -2.59)	-0.74 (-0.76, -0.72)	-59.05 (-61.84, -56.70)
β_2 (GC content)	0.37 (0.36, 0.37)	0.38 (0.37, 0.39)	0.34 (-0.41, 1.27)
β_3 (TEs)	-0.14 (-0.18, -0.11)	0.04 (-0.02, 0.10)	4.76 (2.42, 7.61)
β_4 (Accessibility)	-1.13 (-1.17, -1.09)	0.11 (0.07, 0.15)	-9.98 (-13.51, -5.57)

S2.5 Data analysis of Hi-C and micro-C in human ES cells

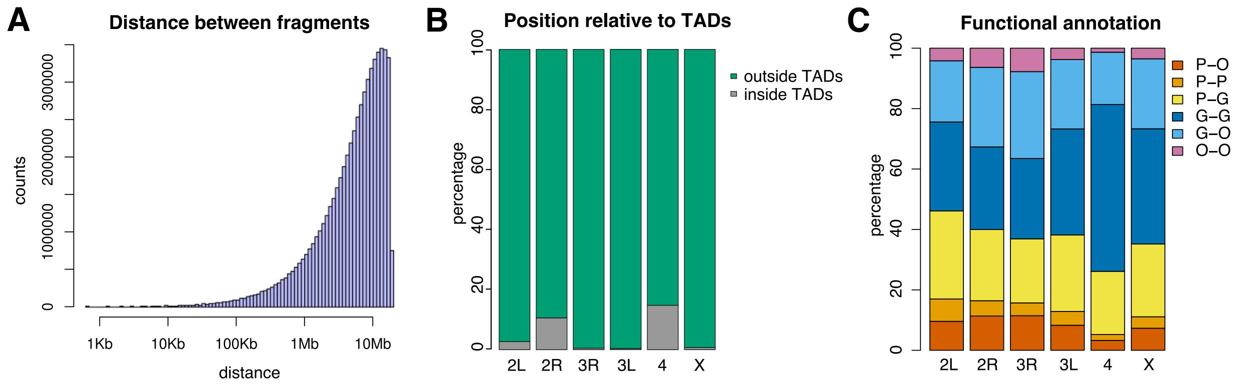

Figure S2: *Genome wide significant interactions in Drosophila Kc167 cells.* (A) Distribution of the genomic distance between the two bins for all significant interactions. (B) classification of significant interactions as either outside TADs when the two bins are located in different TADs or inside TADs when the two bins are located in the same TAD. (C) Percentage of significant interactions that have promoters at one of the bins. We consider the cases of: (P-P) both bins contain promoters, (P-O) only one bins contains promoters and (O-O) none of the bins contain any promoter.

Table S8: Posterior means of our estimated $\vec{\beta}$s for noise, signal and false signal components of Replicate 1 and Replicate 2 of Chromosome 2L respectively. The 95% credible intervals are shown inside the brackets.

Parameters	Posterior mean (noise), k=1	Posterior mean (signal), k=2	Posterior mean (false signal), k=3
β_0 (intercept)	-17.34 (-17.66, -17.02)	6.82 (6.27, 7.45)	499.97 (499.02, 500.96)
β_1 (genomic distance)	-1.77 (-1.80, -1.74)	-0.61 (-0.69, -0.56)	-62.58 (-63.37, -61.70)
β_2 (GC content)	0.33 (0.33, 0.34)	0.34 (0.33, 0.34)	0.34 (0.21, 0.45)
β_3 (TEs)	-0.17 (-0.21, -0.13)	-0.49 (-0.72, -0.29)	4.40 (2.33, 6.75)
β_4 (Accessibility)	-0.53 (-0.56, -0.49)	0.79 (0.68, 0.94)	-5.10 (-6.73, -3.44)
Parameters	Posterior mean (noise), k=1	Posterior mean (signal), k=2	Posterior mean (false signal), k=3
β_0 (intercept)	-17.33 (-17.69, -16.94)	7.03 (6.40, 7.69)	499.98 (499.04, 500.88)
β_1 (genomic distance)	-1.78 (-1.81, -1.74)	-0.62 (-0.70, -0.56)	-64.57 (-65.33, -63.77)
β_2 (GC content)	0.34 (0.33, 0.34)	0.34 (0.33, 0.34)	0.33 (0.22, 0.44)
β_3 (TEs)	-0.18 (-0.21, -0.15)	-0.51 (-0.71, -0.27)	-0.40 (-3.77, 1.64)
β_4 (Accessibility)	-0.53 (-0.56, -0.49)	0.79 (0.64, 0.91)	-0.37 (-1.58, 0.69)

Table S9: Posterior means of our estimated $\vec{\beta}$s for noise, signal and false signal components of human Chromosome 8, region 60M : 70M for data generated using the Hi-C method. The 95% credible intervals are shown inside the brackets.

Parameters	Posterior mean (noise), k=1	Posterior mean (signal), k=2	Posterior mean (false signal), k=3
β_0 (intercept)	1.06 (0.79, 1.49)	5.84 (5.67, 5.99)	5.69 (5.53, 5.79)
β_1 (genomic distance)	0.15 (0.14, 0.17)	-0.99 (-1.00, -0.98)	-0.99 (-1.01, -0.98)
β_2 (GC content)	0.32 (0.32, 0.33)	-0.58 (-0.84, -0.21)	-0.07 (-0.29, 0.18)
β_3 (TEs)	10.00 (9.97, 10.03)	-0.09 (-0.10, -0.07)	-0.1 (-0.13, -0.09)
β_4 (Accessibility)	0.38 (0.35, 0.40)	0.95 (0.92, 0.98)	1.10 (0.97, 1.19)

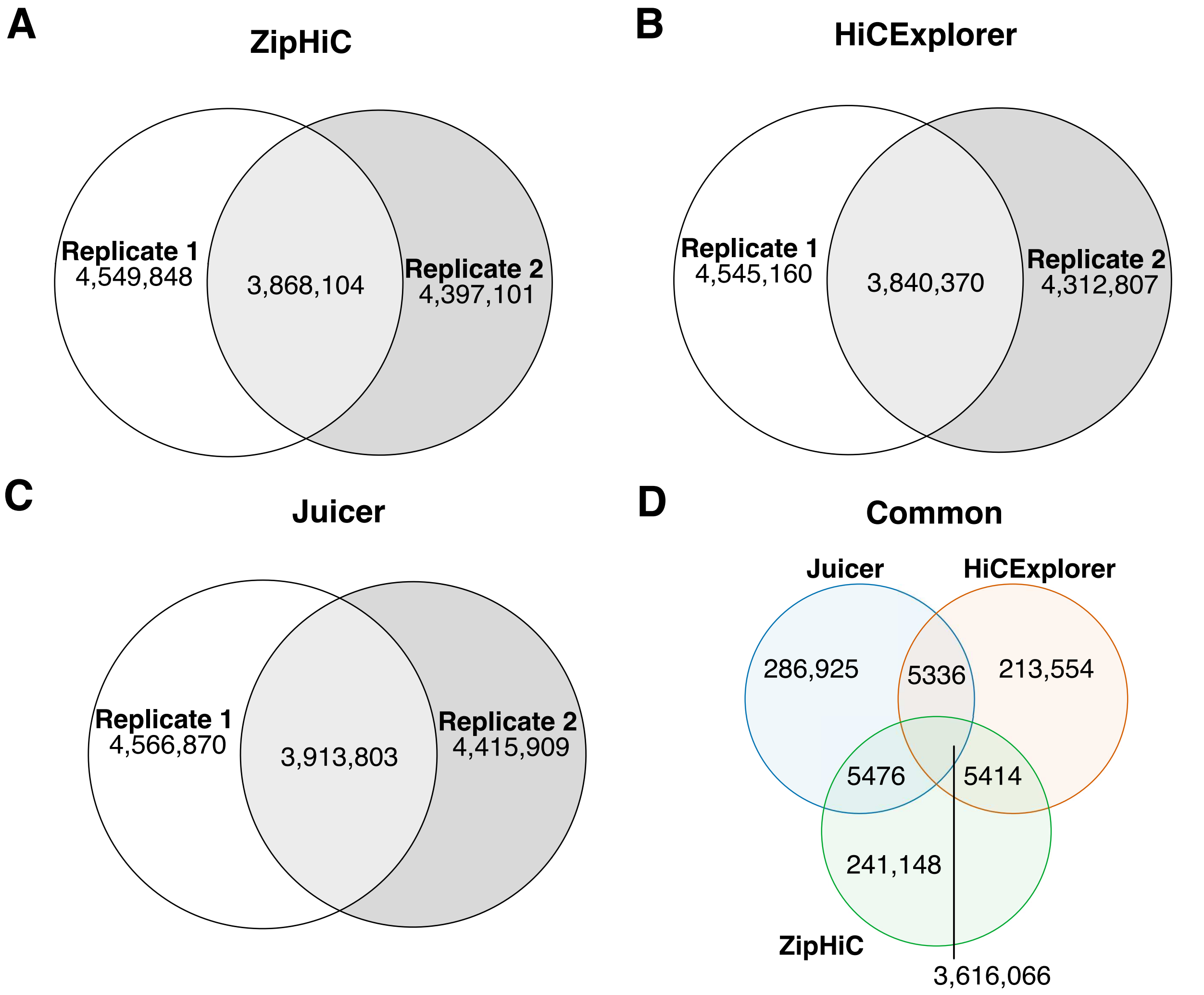

Figure S3: *Comparisons of biological replicates in Drosophila Kc167 cells.* We detected significant interactions in two biological replicates of Drosophila Kc167 cells, using: (A) ZipHiC, (B) HiCExplorer and (C) Juicer. We considered that two interactions detected by the different tools are common if both anchors overlap fully, that is, the start and end of an anchor in one pair matches the start and end of corresponding anchor in the other pair. (D) The overlap of significant interactions detected in both replicates for each method (ZipHiC, HiCExplorer and Juicer). More than 92% of the significant interactions are detected by all three methods.

Table S10: Posterior means of our estimated $\vec{\beta}$s for noise, signal and false signal components of human Chromosome 8, region 60M : 70M for data generated using the micro-C method. The 95% credible intervals are shown inside the brackets.

Parameters	Posterior mean (noise), k=1	Posterior mean (signal), k=2	Posterior mean (false signal), k=3
β_0 (intercept)	0.98 (0.55, 1.58)	7.91 (7.73, 8.09)	8.33 (8.08, 8.53)
β_1 (genomic distance)	0.15 (0.12, 0.17)	-1.40 (-1.43, -1.38)	-1.41 (-1.43, -1.38)
β_2 (GC content)	0.32 (0.32, 0.33)	1.60 (1.14, 1.96)	0.30 (-0.36, 0.98)
β_3 (TEs)	10.00 (9.97, 10.04)	-0.37 (-0.41, -0.33)	-0.37 (-0.40, -0.34)
β_4 (Accessibility)	0.38 (0.35, 0.41)	1.77 (1.58, 1.94)	1.81 (1.71, 1.86)

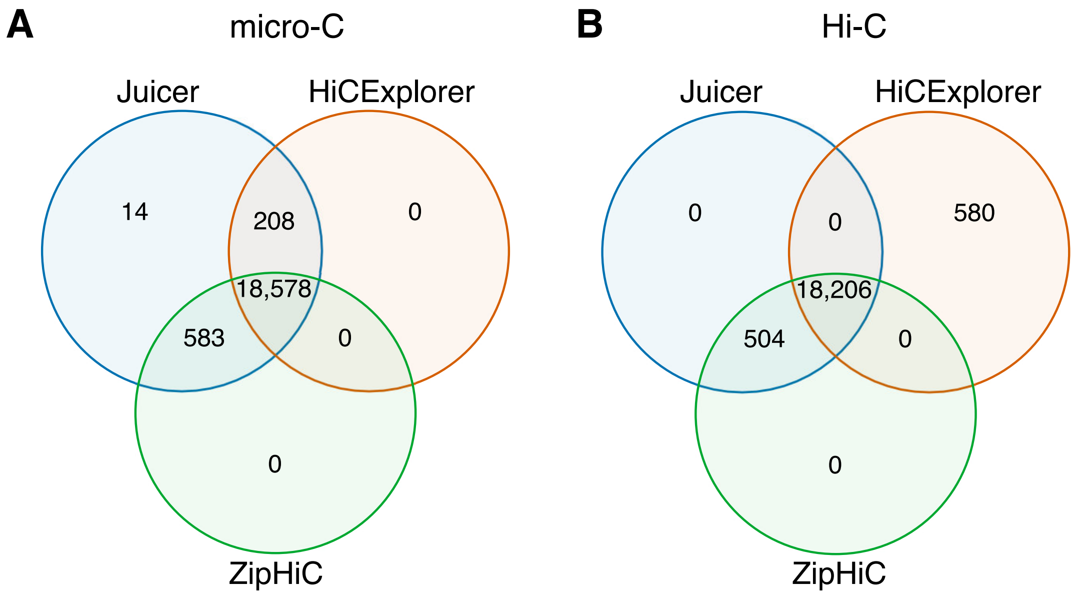

Figure S4: *Comparison between ZipHiC, HiCExplorer and Juicer on human data..* (A) We considered the region 60-70Mb of the human chromosome 8 and data from (A) micro-C and (B) Hi-C in human ES cells. We considered that two interactions detected by the different tools are common if both anchors overlap fully.
